# Accuracy of guided versus freehand implant placement for *in vitro* models using 3D-printed surgical guides

**DOI:** 10.6026/973206300214812

**Published:** 2025-12-15

**Authors:** Dayanand Huddar, Nikil Kumar Jain, Aditya Narayan Shukla, Prashant S Patil, Abhaya Chandra Das, Syed sajid basha

**Affiliations:** 1 Department of Prosthodontics, Bharati Vidyapeeth (Deemed to be University), Dental college and Hospital, Sangli, Maharashtra, India; 2 Department of Oral and Maxillofacial Surgery, Yogita Dental College And Hospital, Khed, Maharashtra, India; 3 Department of Oral & Maxillofacial Surgery, Babu Banarasi Das College of Dental Sciences, Lucknow, Uttar Pradesh, India; 4 Department of Prosthodontics, Yogita Dental College and Hospital, Khed, Maharashtra, India; 5 Department of Periodontics and Oral Implantology, Institute of Dental Sciences, Siksha 'O' Anusandhan (Deemed to be University), Khandagiri Square, Bhubaneswar, Odisha, India; 6 Department of Oral and Maxillofacial Surgery, Maharana pratap college of dentistry and research centre, Gwalior, India

**Keywords:** dental implants, surgical guide, 3D printing, implant accuracy, computer-aided design, freehand placement, *In vitro* study

## Abstract

Proper positioning of implants is necessary in attaining optimum functional and aesthetic results, but freehand methods tend to result in displacement. Therefore, it is of interest to compare the accuracy of 3D-printed surgical guides and free hand placement of implants in polyurethane jaw models. Angular, horizontal and vertical deviation was much lower with guided implants than with freehand placement. Guided surgery also significantly decreased operator variability. Three dimensional printed surgical maps increase the accuracy of implant placement, which justify that they should be used to promote better predictability and clinical performance.

## Background:

Exact positioning of dental implant is the key to the long-term clinical success, the best possible result of prosthetic placement, and patient satisfaction [1]. Proper positioning of the implants should have taken into account various factors such as bone volume, anatomy, the need of the prosthetic and biomechanical principles [2]. Shifts that occur in the intended location of the implant may lead to complication like nerve damage, sinus perforation and poor bone incorporation, complications with the prosthetic and poor aesthetics [3]. There is little advancement in the conventional method of placing freehand implants, as the entire process depends on the experience, clinical judgment and interpretation of two-dimensional radionuclides [4]. Although with skilled hands, the positioning of the implant is very variable in freehand approaches, research has shown that in the complex anatomical cases, there is a high degree of variation in the implant positioning [5]. It is this variation that has led to the creation of guided surgery protocols in order to improve predictability and precision. Implant dentistry has been transformed by computer-aided design and computer-aided manufacturing (CAD/CAM) technologies that have allowed a detailed pre-operative planning and manufacturing of patient-specific surgical guides [6]. These guides are created by different manufacturing approaches such as 3D printing to transfer the virtual plan to the surgical field in a high fidelity way [7]. With the advancement of three dimensional printing technologies, which is now available in the form of stereolithography and selective laser sintering, the use of guided implant surgery has gained broad applicability due to the increasing availability and affordability of this technology [8, 9]. Other meta-analyses also showed greater accuracy of guided than freehand placement, but again there was great heterogeneity across the studies [10]. Nonetheless, the majority of studies have been conducted on individual commercial systems, and there is not much information on a comparison between 3D-print guides and free-hand methods under a controlled laboratory scenario [11]. *In vitro* research has useful information because it could remove confounding factors that exist in clinical scenarios, patient motion, bleeding, and the challenge of the visualization of soft tissues [12]. Human bone simulated polyurethane models provide standardized platforms on comparative accuracy basis [13]. More so, *in vitro* conditions can be rigorously evaluated to assess the performance of surgical guides on a scale of more than a single operator because of its reproducibility [14]. Although there is emerging evidence that guided implant surgery is effective, some questions still remain on the extent of accuracy improvements possible with 3D-printed guides, the repeatability in various positions of implants, and the impact of operator experience [15]. These factors need to be thoroughly understood in order to make clinical decisions that are based on evidence. Therefore, it is of interest to quantitatively assess the precision of dental implant placement with the use of 3D-printed surgical guides compared to the traditional freehand methods in standardized polyurethane jaw models.

## Materials and Methods:

## Study design and sample size:

The comparison was done between two methods of implant placement, the use of 3D -printed surgery guides and the use of the traditional freehand method. The calculation of sample size was done with the G + Power software (version 3.1.9.4) on the basis of expected difference in mean angular deviation of 2.5 with a standard deviation of 1.5, 0.05, and 0.90, which gives a minimum size of 15 models per group. To cover the possible errors of the experiment, there were 20 models within each group.

## Model Preparation:

Forty brother polyurethane jaw models (Sawbones, Vashon Island, WA, USA) of partially edentulous mandibles were utilized. The models were made by cortical shell (1.5 mm thick) over a trabecular bone substitute material with density of 20 pounds per cubic foot (PCF) which is Type II bone quality based on Lekholm and Zarb classification. In the posterior area, each of the models had three edentulous sites that were at positions 36, 37 and 46.

## Virtual planning and guide fabrication:

Cone-beam computed tomography (CBCT) scanning (NewTom VGi, Verona, Italy) of all models was performed at 110 kV, 8 mA, voxel size 0.15 mm and scan time 18 seconds. The DICOM files were brought into an implant planning software (Blue Sky Plan 4.0, Blue Sky Bio, Grayslake, IL, USA). Three virtually planned implants (titanium grade 4) were 4.0 x 10 mm each and placed on a standardized position relative to each other, parallel to the occlusal plane, 3 mm between the implants and the adjacent teeth, and 2 mm between the implants and vital structures. In the case of the guided group, the sleeves of the surgical guides were made three-point supported (tooth-supported design) and with a diameter of 4.8 mm. The guides were additively produced with the help of a stereolithography 3D printer (Form 3B, Formlabs, Somerville, MA, USA) and surgical guide resin (clear, biocompatible). The final processing after printing was done by washing 20 minutes with isopropyl alcohol, air drying and UV curing 60 minutes at 60°C. The guides were press-fitted with metal guides of the specifications.

## Allocation and randomization:

The guided group or the freehand group had models randomly assigned using a computer-generated randomization (www.randomizer.org). The models were assigned their own identification number and group assignment was hidden in their sealed envelopes until the surgery period.

## Surgical Procedures:

All the procedures were done by three experienced oral surgeons (more than 10 years of experience with implants, over 500 implants placed). The implants were inserted by each surgeon on an equal number of both groups to eliminate the possibility of operator variability. The surgeons were oriented with the models and equipment by pilot procedures of different models which were not the subject of the analysis. In the guided group, surgical guide was placed on the models and held manually as the models were being drilled. Sequential drilling was done in a guided surgical kit through 2.0 mm pilot drill, 2.8 mm intermediate drill, and 3.5 mm final drill which were through the guide sleeves. With the handpiece, a torque-controlled handpiece, 35 Ncm was placed to put the implants (4.0 10 mm) at the desired depth. In the freehand group, the surgeons were provided with printed copies of the virtual plan with the perfect implant positions and angulations. Their traditional protocols of drilling followed manual visual and tactile feedback. The guided group had the same sequence of drills and implants. Each of the procedures was carried out in a dental simulator unit in the standardized lighting conditions. Both groups were drilled using irrigation with saline solution.

## Accuracy assessment:

Post-operative CBCT scans were acquired using identical parameters as pre-operative scans. DICOM files were imported into the planning software, and superimposition of pre-operative and post-operative scans was performed using surface matching algorithms. The achieved implant positions were compared to the planned positions.

## Primary outcome variables measured were:

[1] Angular deviation: angle between the planned and actual implant axis

[2] Horizontal deviation at entry point: 3D distance between planned and actual implant positions at the coronal aspect

[3] Horizontal deviation at apex: 3D distance between planned and actual implant positions at the apical aspect

[4] Vertical depth deviation: difference in vertical position of implant platform

All measurements were performed by a single calibrated examiner blinded to group allocation. Intra-examiner reliability was assessed by repeating measurements on 20% of samples after two weeks.

## Statistical analysis:

Data were analyzed using SPSS Statistics version 27.0 (IBM, Armonk, NY, USA). Normality was assessed using Shapiro-Wilk tests and Q-Q plots. Continuous variables were expressed as mean ± standard deviation. Independent t-tests were used to compare deviations between guided and freehand groups. One-way ANOVA was performed to evaluate inter-operator differences. Intra-class correlation coefficient (ICC) was calculated for reliability assessment. Statistical significance was set at p<0.05.

## Results:

All 40 models were successfully completed without technical failures. A total of 120 implants were placed and analyzed (60 in each group). Post-operative CBCT image quality was adequate for superimposition and measurement in all cases. Intra-examiner reliability demonstrated excellent agreement (ICC = 0.94, 95% CI: 0.89-0.97). Table 1 (see PDF) presents the overall comparison of accuracy between guided and freehand implant placement. The guided group demonstrated significantly lower angular deviation (2.8 ± 1.2°) compared to the freehand group (5.9 ± 2.4°; p<0.001), representing a 52.5% reduction in angular error. Horizontal deviation at the entry point was significantly reduced in the guided group (0.6 ± 0.3 mm) versus freehand (1.8 ± 0.9 mm; p<0.001), a 66.7% improvement. At the apex, horizontal deviation was 0.9 ± 0.4 mm for guided placement compared to 2.4 ± 1.1 mm for freehand (p<0.001), representing a 62.5% reduction. Vertical depth deviation was significantly lower with guided placement (0.4 ± 0.2 mm) than freehand (1.2 ± 0.6 mm; p<0.001). Analysis by implant position revealed consistent superiority of guided placement across all three sites (Table 2 - see PDF). For position 36, angular deviations were 2.6 ± 1.0° (guided) versus 5.5 ± 2.2° (freehand; p<0.001). Position 37 showed deviations of 3.0 ± 1.3° versus 6.2 ± 2.5° (p<0.001), and position 46 demonstrated 2.8 ± 1.2° versus 6.0 ± 2.5° (p<0.001). Similarly, horizontal deviations at both entry and apex points were significantly lower for guided placement across all positions (p<0.001 for all comparisons). Table 3 (see PDF) presents the comparison of accuracy among the three operators. For the guided group, no significant differences were found among operators for any accuracy parameter (angular deviation: p=0.342; horizontal deviation entry: p=0.428; horizontal deviation apex: p=0.391; vertical deviation: p=0.456). In contrast, the freehand group demonstrated significant inter-operator variability for angular deviation (p=0.021) and horizontal deviation at apex (p=0.036), though not for entry point deviation (p=0.089) or vertical deviation (p=0.127).

In the guided group, 91.7% (55/60) of implants achieved angular deviations ≤4°, compared to 43.3% (26/60) in the freehand group. For horizontal deviation at entry point, 95.0% (57/60) of guided implants had deviations ≤1.0 mm versus 38.3% (23/60) for freehand. At the apex, 93.3% (56/60) of guided implants showed deviations ≤1.5 mm compared to 35.0% (21/60) for freehand. All guided implants (100%) achieved vertical deviations ≤1.0 mm, while only 61.7% (37/60) of freehand implants met this criterion. Maximum deviations observed were: angular 5.8° (guided) vs 11.2° (freehand); horizontal entry 1.4 mm vs 4.2 mm; horizontal apex 2.1 mm vs 5.3 mm; and vertical 0.9 mm vs 2.8 mm.

## Discussion:

This *in vitro* experiment showed that 3D-printed surgical guides demonstrated a high level of accuracy in dental implant positioning in comparison to freehand methods in all the measured parameters. The guided method decreased angular deviation by about 53, horizontal deviations by 63-67 and vertical deviation by 67. These results confirm a non-acceptance of the null hypothesis and are in line with the current results that indicated the utility of guided surgery [16, 17]. The average angular deviation of 2.8°, which was the average of 2.0° to 4.5° in systematic reviews, of guided surgery, as observed in the current study is 2.8°. The mean angular deviation in the freehand group of 5.9 is also congruent with laboratory reports of 4.5 to 8.0 angular deviations [18]. Angular precision is clinically important as the deviations may affect the results of the prosthetic, especially in the implant-supported fixed prostheses where parallel implant positioning enables the optimal distribution of loads and is easy to retrieve [19]. The horizontal errors at the starting point (0.6 mm guided vs 1.8 mm free hand) and apex (0.9 mm vs 2.4 mm) as realized in this study reflect a significant clinical implication. Excessive deviation at the apex above 2 mm can predispose to injury to normal anatomical structures of the inferior alveolar nerve, mental foramen or other adjacent tooth roots [20]. Even smaller deviations in the anterior maxilla can interfere with the aesthetics of outcomes in terms of emergence profile and soft tissue architecture [21]. The fact that the guided method keeps deviations to less than 1 mm at the entry point in 95 percent of the cases is a great safety and predictability benefit. Vertical depth control is also crucial to the proper positioning of the prosthetic platform and adequate biological width [22]. The much less vertical deviation using guided positioning (0.4 mm vs 1.2 mm) indicates that surgical guides are effective in standardization of the depth of drilling, which may decrease the chances of implant malpositioning that may lead to revision surgery or impairment of the aesthetics [23]. An interesting observation was that the inter-operator error decreased significantly when the placement was guided. Although the free hand group recorded a great variation between experienced surgeons, the guided group was consistent in the accuracy according to operator. This observation implies that the surgical guides can be used to standardize the results, which would be beneficial to less trained clinicians or people switching to digital flows [24]. It must be noted, however, that the study utilized only experienced implantologists as the operators; this may not be the same with new surgeons. The precision of the 3D-printed guides in the study is similar to systems commercially sold under guided surgery even though it may be less expensive [25]. This result can be of significance to clinical practice accessibility especially in areas where costly proprietary systems can be prohibitive. Nevertheless, the precision of 3D-printed guides relies on various variables such as the printer resolution, the material characteristics, post-processing mechanisms, and surgical procedure [26]. The high accuracy of guided placement may be caused by several factors. Mechanical constriction of drill movement surgical guides is used to provide three-dimensional movement of the drills with no visual estimation and manual dexterity when placed freely [27]. The guide sleeves offer sustained stabilization of the drills during the preparation sequence and reduce the lateral drift which usually happens with handheld methods [28]. Also, guides do not require real-time interpretation of spatial orientation that is especially very difficult in situations where two-dimensional radiographs are used [29]. In spite of these benefits, there are disadvantages of guided surgery. Guide fabrication takes more time, money and technical skills [30]. Guidance in surgery can reduce visibility and access to irrigation and, therefore, can increase friction and heat production [31]. In addition to this, the guides created on pre-operative scans do not adapt to intraoperative observations which may induce a change in position [32]. Guides are also rigid, which implies that the errors made in the process of planning are directly passed to the surgical field and the importance of careful virtual planning cannot be overstated [33]. This paper has a number of limitations. *In vitro* design, albeit offering accurate assessment of the conditions, cannot be a full replica of the clinical situation with tight mouth opening, soft tissues, bleeding, patient movement, and saliva control [34]. Polyurethane models though simulating bone density are not as heterogeneous as bone is, and do not contain anatomical features such as neurovascular bundles to aid surgical decision-making [35]. The tooth-supported guide, described in this study, might not be applicable in the fully edentulous cases where the bone-supported or mucosa-supported guide would be required which may affect the accuracy in a different way [36]. Also, the implant system or size considered in the study is single and hence cannot be generalized to other systems and dimensions. The future studies will examine the accuracy of guided surgery in clinical practice involving a non-homogeneous patient group, consider the effectiveness of various forms of guide support, compare the results of different types of 3D printing technologies and materials, analyze long-term 3D printing prosthetic and biological results, and explore cost-effectiveness in a variety of clinical settings [37]. Investigations that integrate artificial intelligence and machine learning to maximize the design of the guide and forecast the accuracy can further benefit guided surgery protocols [38].

## Conclusion:

The accuracy of surgical guide placement with use of 3-D printing of surgical guides significantly reduces angular, horizontal and vertical deviations in dental implant insertion, as opposed to freehand techniques. The guided approach ensures less variability of the operators too, which enhances predictability and uniformity. Such findings favor the clinical application of 3D-printed guides to obtain a more accurate and reliable implant outcome.
